# Clinical and Analytical Validation of Two Methods for Ki-67 Scoring in Formalin Fixed and Paraffin Embedded Tissue Sections of Early Breast Cancer

**DOI:** 10.3390/cancers16071405

**Published:** 2024-04-03

**Authors:** Snežana Đokić, Barbara Gazić, Biljana Grčar Kuzmanov, Jerca Blazina, Simona Miceska, Tanja Čugura, Cvetka Grašič Kuhar, Jera Jeruc

**Affiliations:** 1 Department of Pathology, Institute of Oncology, 1000 Ljubljana, Slovenia; sdokic@onko-i.si (S.Đ.); bgazic@onko-i.si (B.G.);; 2 Faculty of Medicine Ljubljana, University of Ljubljana, 1000 Ljubljana, Slovenia; smiceska@onko-i.si; 3 Department of Cytopathology, Institute of Oncology, 1000 Ljubljana, Slovenia; 4 Institute of Pathology, Faculty of Medicine, University of Ljubljana, 1000 Ljubljana, Slovenia; 5 Department of Medical Oncology, Institute of Oncology Ljubljana, 1000 Ljubljana, Slovenia

**Keywords:** breast cancer, immunohistochemistry, Ki-67, MIB-1, S-phase fraction, proliferation

## Abstract

**Simple Summary:**

Ki-67, a protein found in actively growing and dividing cancer cells, serves as an important tumor biomarker in breast cancer. A higher Ki-67 level indicates faster cell multiplication. In this study, pathologists evaluated Ki-67 using two methods: a visual whole-slide assessment and a quantitative tumor margin analysis. The study was performed in early-stage breast cancer patients. The findings revealed that Ki-67 is a reliable method for guiding clinical decision-making. Notably, a cut-off value of 20% appeared most appropriate for prognosis estimation and prediction of therapy benefit in our cohort of patients.

**Abstract:**

Proliferation determined by Ki-67 immunohistochemistry has been proposed as a useful prognostic and predictive marker in breast cancer. However, the clinical validity of Ki-67 is questionable. In this study, Ki-67 was retrospectively evaluated by three pathologists using two methods: a visual assessment of the entire slide and a quantitative assessment of the tumour margin in 411 early-stage breast cancer patients with a median follow-up of 26.8 years. We found excellent agreement between the three pathologists for both methods. The risk of recurrence for Ki-67 was time-dependent, as the high proliferation group (Ki-67 ≥ 30%) had a higher risk of recurrence initially, but after 4.5 years the risk was higher in the low proliferation group. In estrogen receptor (ER)-positive patients, the intermediate Ki-67 group initially followed the high Ki-67 group, but eventually followed the low Ki-67 group. ER-positive pN0-1 patients with intermediate Ki-67 treated with endocrine therapy alone had a similar outcome to patients treated with chemotherapy. A cut-off value of 20% appeared to be most appropriate for distinguishing between the high and low Ki-67 groups. To summarize, a simple visual whole slide Ki-67 assessment turned out to be a reliable method for clinical decision-making in early breast cancer patients. We confirmed Ki-67 as an important prognostic and predictive biomarker.

## 1. Introduction

Breast cancer is the most common cancer in women worldwide, accounting for 24% of all cancers, and is the leading cause of cancer-related deaths in women [1]. In Slovenia, the crude incidence of breast cancer in 2016–2020 was 142.2, and the mortality rate was 42.1 per 100,000 women [2]. 

Adjuvant systemic therapy aims to reduce the risk of disease recurrence and mortality. To assess the risk of recurrence, we use a variety of tools. In addition to the classic prognostic factors such as tumour and nodal stage, grade, and estrogen receptor (ER) and human erbB-2 (HER2) status, the proliferative activity of a tumour provides unique additional information. Proliferation is one of the hallmarks of cancer [3]. It can be assessed using various methods: by counting mitoses on tissue sections, by estimating the percentage of cells in the S-phase of the cell cycle (S-phase fraction; SPF) using flow cytometry, by detecting antigens (e.g., Ki-67) associated with proliferation by immunohistochemistry (IHC) and, in recent years, by checking for ER-positive tumours using genomic tests that include the expression of a number of proliferation genes (Oncotype Dx, Genomic Grade Index, and PAM50) [4,5,6,7]. However, in routine practice, the detection of Ki-67 (MIB-1 clone) on primary carcinoma tissue via IHC is most commonly used to assess proliferative activity and to predict which patients will benefit from chemotherapy. 

Ki-67 is a nuclear protein that is expressed in proliferating cells, but is not present in quiescent (G0) cells. It can be easily detected through IHC in tissue samples [5]. Tumour proliferation activity, as assessed by Ki-67 levels, has been investigated as a prognostic and predictive factor in several studies [8,9,10,11]. After the discovery of different gene expression patterns in breast cancer led to a classification into molecular subtypes, Ki-67 became an important biomarker to differentiate between luminal A and luminal B subtypes (cut-off < 14%) [9]. This is especially important because luminal B tumours have a worse prognosis for recurrence and death compared to luminal A tumours [9]. IHC surrogates for molecular subtypes of breast cancer were adopted by the St. Gallen consensus in 2011 [12]. Later, a majority of the St. Gallen Expert panel voted that a threshold of ≥20% was indicative of ‘high’ Ki-67 status and aggressive tumour behaviour, independently of molecular carcinoma type [13]. Meta-analysis performed by de Azambuja et al. showed that high Ki-67 (cut-off range 5–30%) leads to lower disease-free survival (DFS) and overall survival (OS) in the overall population, as well as in node-negative and node-positive subgroups [14]. In addition, Yerushalmi et al. showed that in the neoadjuvant setting, high Ki-67 can predict the response to chemotherapy, and low Ki-67 is more likely to indicate the benefit of endocrine therapy [8].

In the past, proliferative activity in breast carcinomas was often determined via SPF, a quantitative, rapid, reliable and inexpensive flow-cytometric method for estimating cell proliferation and DNA ploidy. In our previous work on a cohort of 770 breast cancer patients, we showed that 43% had diploid tumours, and the median SPF was 6.7%. A high SPF (≥6.7%) was an independent unfavourable prognostic factor for DFS and OS [15], consistent with the findings in the review published by Colloza et al. [16]. In most studies, a good correlation between SPF and Ki-67 has been found [17,18,19,20,21,22,23], although in others this was not confirmed [24,25].

Despite the robust correlation of Ki-67 with prognosis and the benefit of systemic therapy, Ki-67 is still not uniformly recommended as a biomarker in breast cancer. The main issue preventing its recommendation is the lack of accurate and reproducible reporting of Ki-67. To standardize the procedures for Ki-67 assessment in breast cancer, the International Ki-67 in Breast Cancer working group (IKWG) published a first recommendation in 2011 aiming to minimize the pre-analytical and analytical variability and to harmonize scoring methods and interpretation for Ki-67 [26]. The recommendations were last updated in 2021 [27]. In the meantime, the IKWG has conducted two studies to assess the concordance of Ki-67 scoring [28,29]. While the intra-laboratory reproducibility was high, the inter-laboratory concordance was only moderate. It is believed that the clinical validity of Ki-67 assessment for clinical decision-making is not yet optimal, especially with regard to an accurate Ki-67 cut-off value associated with prognosis and treatment planning [30]. In the updated IKWG guidelines, no clear cut-off value between low and high Ki-67 could be defined; therefore, two cut-off values were recommended: ≤5% for a low value, and ≥30% for a high value [27,30]. On the other hand, the recommendations of the European Society for Medical Oncology (ESMO) differ slightly from those of the IKWG with regard to low proliferation; in the ESMO recommendations, all tumours with Ki-67 ≤ 10% are considered to be clearly low proliferative, and those with Ki-67 ≥30% highly proliferative [31]. Ki-67 proliferative activity is currently not included in the recommendations for the diagnosis and treatment planning of breast cancer by the American Society for Clinical Oncology (ASCO) or the National Comprehensive Cancer Network (NCCN).

Our pathology department at the Institute of Oncology Ljubljana has many years of extensive experience with both methods for determining proliferative activity (Ki-67 and SPF). Here, we conducted a retrospective study on breast cancer patients with a long follow-up period of almost 30 years in which proliferative activity was determined using both methods. We investigated the agreement and correlation of two different Ki-67 scoring methods and used the quantitative SFP method as a control. First, we assessed the inter-observer agreement of each Ki-67 scoring method. Second, we evaluated the agreement and correlation between the Ki-67 methods and the SPF. Finally, we assessed the prognostic and predictive value of Ki-67 and SPF in the overall cohort and in the ER-positive cohort. We hypothesized that both Ki-67 scoring methods would indicate very good inter-observer agreement, as well as good agreement and correlation with SFP, and that Ki-67 could be an independent prognostic factor to predict survival and the benefit of systemic therapy in breast cancer patients.

## 2. Materials and Methods

### 2.1. Patients and Treatment 

Patients diagnosed for early breast carcinoma at the Institute of Oncology Ljubljana, Slovenia, in years 1992–1997, were included in our retrospective study. Data on patients, their tumour characteristics (tumour size, histology type, grade, lymphovascular invasion, axillary node involvement and ER status), and treatment were retrieved from patients’ medical records. The ER status was, at the time of diagnosis, determined biochemically [32], using 10 fmol/mg proteins as a cut-off value for positivity. Data on HER2 status was not available, as it was not part of the standard procedure at that particular time.

Inclusion criteria were age ≥ 18 years, stages IA to IIIB, undergoing primary surgical treatment, and having data on SPF and available archival formalin-fixed paraffin-embedded (FFPE) tissue specimens. Exclusion criteria included carcinoma in situ, metastatic disease, neoadjuvant systemic treatment, no archival FFPE specimens, or technical issues with IHC analysis.

The study was approved by The Slovenian Medical Ethics Commission (No. 0120-176/2019-4). All procedures during the study were carried out following the Declaration of Helsinki and Good Clinical Practice.

### 2.2. Pathohistological Examinations: IHC Staining and Scoring for Ki-67

We followed criteria for biomarker studies (The Biospecimen Reporting for Improved Study Quality (BRISQ) [33] and REMARK criteria [34]).

An IHC examination was performed on archival FFPE surgical excision specimens of primary tumours fixed in 10% neutral buffered formalin for 8 to 72 h using the fully automated IHC staining system Ventana BenchMark ULTRA (Ventana, Roche Diagnostics, Tucson, AZ, USA), for which 2–4 µm thick tissue sections were freshly cut and dried at 56 °C for 2 h. Heat-mediated epitope retrieval was performed with Cell Conditioning Solution 1 (Ventana, Roche Diagnostics, Tucson, AZ, USA; cat. No. 950-124) for 88 min at 100 °C. The retrieved epitope was detected using monoclonal anti-Ki-67 antibody (Agilent, Santa Clara, CA, USA, cat No M7240; clone MIB-1; diluted at 1:200). Sections were incubated on board for 60 min at 37 °C, visualized using the three-step multimer detection system OptiView DAB IHC Detection Kit (Ventana ROCHE Inc., Tucson, AZ, USA; cat. No. 760-700) according to the manufacturer’s instructions, and counterstained with haematoxylin (cat. No 790-2208; Ventana Medical Systems, Roche Diagnostics, Tucson, AZ, USA). The performance of the IHC reaction was monitored using in-house on-slide controls. The control sample was composed of cells from a normal appendix, normal cervix, and three breast tumours with various levels of Ki-67 positive tumour cells. The Ki-67 IHC protocol has passed external quality assessment in several programs (UK NEQAS, NORDIQC, and LabQuality) with excellent scores.

**Figure 1:**
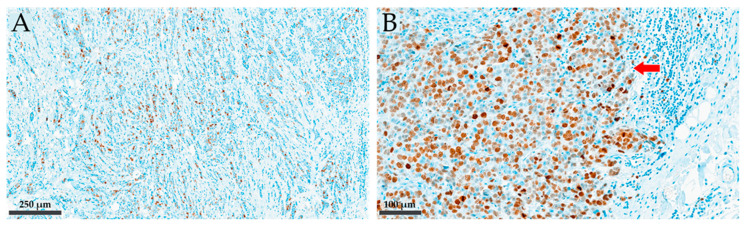
Illustration of the two different Ki-67 assessment methods applied in this study. (**A**) Method 1: semi-quantitative assessment on the whole tumour slide at 100× magnification; (**B**) Method 2: quantitative assessment on tumour edge at 200× magnification (presented with the arrow).

Three pathologists, two specialized in breast pathology (SD, BGK), and one pathology resident (TČ) interpreted and scored Ki-67 IHC staining slides independently using two different scoring methods, based on the area of the slide read by light microscopy. In Method 1, the entire tumour slide was assessed, and the average score was determined semi-quantitatively as a percentage of positively stained tumour cell nuclei at 100× magnification to 1% accuracy. In Method 2, only the tumour edge was assessed, and the average score was determined quantitatively by counting positively stained tumour cell nuclei among 500 counted tumour cells in five consecutive fields of view of 200× magnification (100 cells in each field of view). Demonstration of the two different Ki-67 visual scoring methods (semi-quantitative on the whole tumour slide, and quantitative on the tumour edge) is provided in Figure 1.

### 2.3. Flow-Cytometric Determination of S Phase Fraction

SPF was determined using a flow-cytometric method, as previously reported by our group [15]. Briefly, fine needle aspiration biopsy samples (FNAB) of patients with invasive breast carcinomas included in our study were prepared according to the standard protocol proposed by Otto et al. [35] and implemented at our hospital for FNAB samples according to the guidelines published by Pogačnik et al. [36]. The samples were acquired using a PAS II Flow Cytometer (Partec, Münster, Germany). From each sample, 5000–65,000 signals were recorded at a flow rate of about 200 counts per second, and a 1024-channel DNA histogram was generated. Before sample acquisition, the instrument was set up using trout erythrocytes and human lymphocytes to determine the diploid area and adjust coefficient of variation (CV) of the G0/G1 peak to 1.5–2%. The SPF of the diploid tumours was calculated as the number of signals in the interval between the G0/G1 and G2/M peaks (S-phase region of the histogram) divided by the sum of all recorded signals in the G0/G1 peak, G2/M peak, and interval of the S-phase region. The SPF of aneuploid tumours was calculated in a similar way, considering only the number of signals in the aneuploid cell cycle.

### 2.4. Data Analysis

Numerical variables were presented as medians and interquartile ranges (IQRs), and categorical variables as frequencies and percentages. The distribution of SPF and Ki-67 values was presented graphically using boxplots. Ki-67 was log transformed because its distribution was non-symmetrical [37]. The differences between groups were tested with the Pearson chi-square test or Fisher’s exact test for categorical data and the unpaired Student’s *t*-test for continuous data. A *p* value of less than 0.05 was considered statistically significant. No adjustments for multiple comparisons were made.

The interrater agreement of Ki-67 scoring between individual pathologists was assessed using the intra-class correlation coefficient (ICC) [38]. ICC estimates and their 95% confident intervals were calculated with SPSS v.22 based on mean-rating (k = 3), absolute agreement, two-way random effects. ICC values < 0.5 indicated poor reliability, 0.5–0.75 moderate reliability, 0.75–0.90 good reliability, and values >0.90 excellent reliability.

To visually determine the agreement and to identify any systematic bias between different methods, the Bland-Altman difference plot was used for proliferation assessment (SPF and Methods 1 and 2 for Ki-67) [39]. 

The relationship between SPF and the two methods for Ki-67 was presented in scatterplot graphs. The correlation between the IHC methods for Ki-67 and SPF was determined by Kendall’s correlation coefficient analysis, which is more robust than the Spearman coefficient and is appropriate for samples with outliers. Kendall’s correlation coefficient was based on pairwise complete observations with the corresponding 95% confidence intervals (CI) using 10^4^ bootstrapped samples. A Kendall’s correlation coefficient of 0.26–0.48 indicates a moderate correlation, and values of 0.49–0.70 constitute a strong positive correlation [40]. 

Time to recurrence (TTR), recurrence-free survival (RFS), and overall survival (OS) curves were estimated using the Kaplan-Meier method. TTR, RFS, and OS were defined as time from surgery to recurrence (TTR), recurrence or death (whichever comes first) for RFS, and death from any reason (OS). Patients without the event were censored at the last observation. To evaluate the prognostic value of Ki-67, the first method (M1) was chosen, since it is used in our daily practice. To evaluate the clinical validity of Ki-67 we used cut-offs as recommended by IKWG [27] (Ki-67 ≤ 5%: low; Ki-67 ≥ 30%: high; Ki-67 6–29%: intermediate), along with ESMO guidelines and the St. Gallen consensus (Ki-67 ≤ 10%: low; Ki-67 ≥ 30%: high, Ki-67 11–29%: intermediate) [12,13,31]. For SPF evaluation, a value of 6.7% was used, based on our previous study [15]. All clinically relevant clinicopathological parameters were included in the survival analysis. A log-rank test was used for comparison of Kaplan-Meier curves. A Cox proportional hazard model and time dependent Cox model were used to assess the prognostic value of studied factors. 

The statistical analysis was performed with the software package ‘R for Windows v2022.07.01, Excel for Windows, and SPSS v.22.

## 3. Results

### 3.1. Patients

**Figure 2:**
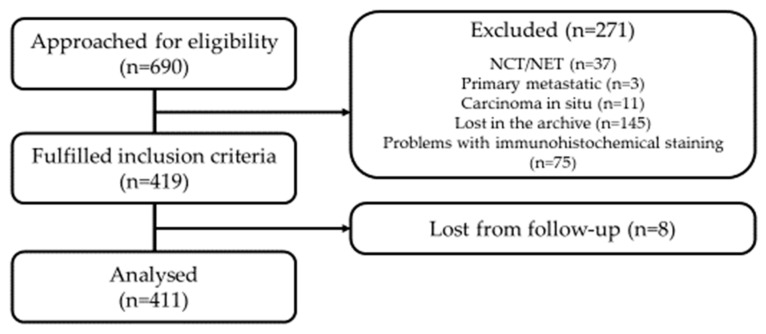
Consort diagram of patients included in the study. NCT: neoadjuvant chemotherapy; NET: neoadjuvant hormone therapy.

Six hundred and ninety patients with invasive breast carcinoma, who were treated between 1992 and 1997 at the Institute of Oncology Ljubljana, were included in our retrospective study. Of those, 411 met the inclusion criteria (Figure 2).

Patients and tumour characteristics are presented in Table 1. All patients were female, the median was age 58.9 years (interquartile range 46.7–67.2), and 70.1% of them were ≥50 years old. The majority of patients (350; 85.2%) had invasive carcinoma of no special type (NST), with 51 (12.4%) of the carcinomas being of the invasive lobular type and 10 (2.4%) of other rare subtypes. Almost half of the patients (48.4%) had grade 3 carcinomas, and only 10.2% had grade 1 carcinomas, according to the Nottingham Grading System. More than half of the patients had tumour stage 2 (53.8%) and negative axillary nodes (55.2%), and 68.7% of the patients had ER+ tumours. HER2 status was unavailable at that time.

### 3.2. Ki-67 Scoring Results

**Figure 3:**
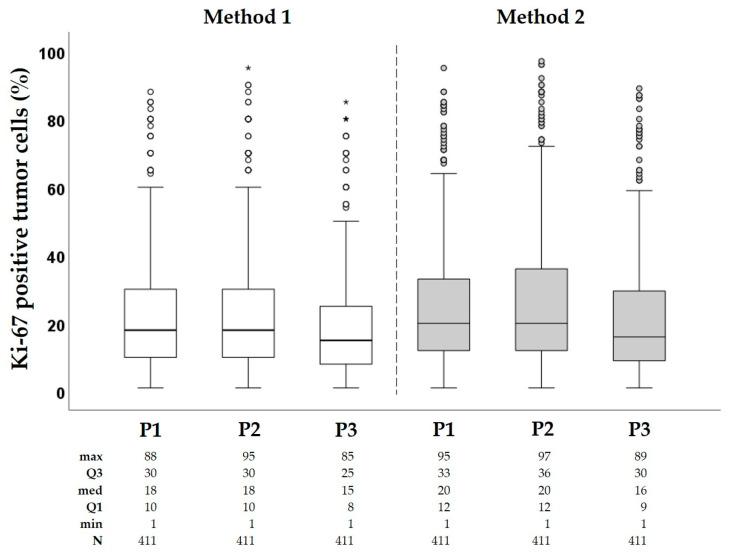
Box-plots of Ki-67 distribution. The bottom and the top of the box in each boxplot represent the first (Q1) and third (Q3) quartiles, and the horizontal line inside the box represents the median. The whiskers extend to minimum and maximum values within 1.5 times the interquartile range from the first and third quartiles. Any data not within the two bars are outliers or extreme outliers and are represented with circles and stars, respectively. P2, P2, P3: pathologist 1, 2, 3, respectively.

Results of Ki-67 assessment by three independent pathologists using both methods (Method 1 and Method 2) are presented in Figure 3. The average Ki-67 score using Method 1 was significantly lower than that obtained using Method 2 (*p* = 0.02; 22% vs. 25.1%, respectively). The median Ki-67 was 16.3% (Q1 9.3, Q3 28.3) and 18.7% (Q1 11.3, Q3 32.7), for Method 1 and Method 2, respectively. Data on Ki-67 were not normally distributed. For parametric testing, we performed log2-transformation (Table S1).

#### 3.2.1. Agreement and Correlation for Ki-67 Scoring

**Figure 4:**
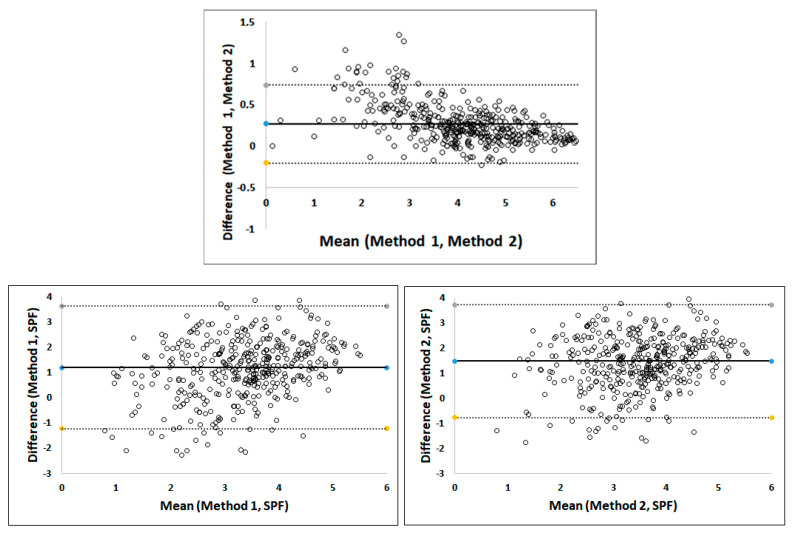
Bland–Altman plots for testing the agreement between Method 1 (M1), Method 2 (M2), and S phase fraction (SPF), performed on log2 transformed data. The *y*-axis represents the difference and the *x*-axis the mean between the two evaluated methods (M1 and M2; M1 and SPF; M2 and SPF). The middle black line represents the average of the differences across all observations. The upper dashed line represents the upper limit of agreement, and the lower dashed line the lower limit of agreement. For each method of Ki-67 assessment, the average value of all three pathologists was used. Blue dots of the Bland–Altman plots present the median differences, while yellow and grey dots represent the lower and upper limits, respectively.

The agreement between all three pathologists for each Ki-67 method was evaluated separately using intraclass correlation (Table 2). For Method 1, the agreement was excellent (ICC higher than 0.90, and the 95% CI did not exceed 0.90). For Method 2, the agreement was good to excellent (ICC > 0.90, but the lower bound of CI was below 0.90). It can be concluded that the intra-laboratory reproducibility was very high, indicating almost perfect agreement between pathologists. The agreement between Method 1 and Method 2 is visualized by a Bland-Altman difference plot (Figure 4, lower left). The bias is low, and the limits of difference are narrow. However, the results of the two methods did not agree well at low mean scores, which means that the two methods cannot be considered interchangeable. Nevertheless, there was a very good correlation between the two methods (Kendall correlation coefficient 0.92 (95% CI 0.91–0.93, *p* < 0.0001)).

#### 3.2.2. Agreement and Correlation between Ki-67 and SPF

**Figure 5:**
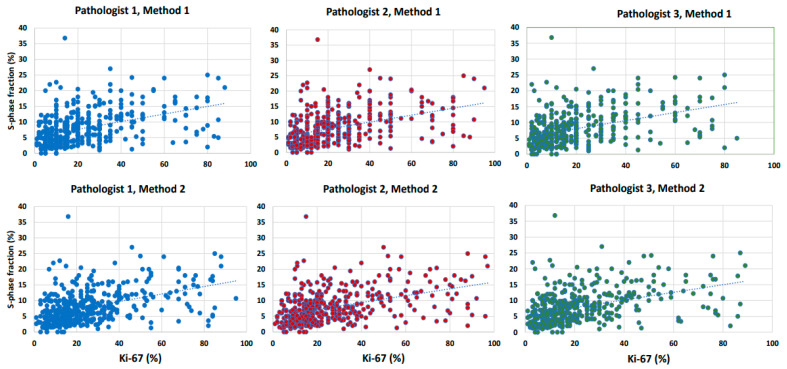
Scatterplots of the relation between each of the three different methods of Ki-67 scoring with the S-phase fraction method. M1: Method 1, M2: Method 2 (continuous values of results).

The Ki-67 values for Methods 1 and 2 reported by each of the three pathologists in relation to SPF are shown in the scatter plot in Figure 5 (upper part for Method 1, lower part for Method 2). The Ki-67 value for a given sample was expectedly higher than the SPF value, because the proportion of proliferating cells is typically higher than the proportion of cells in the S phase. We tested the agreement between SPF and Ki-67 assessment with Bland–Altman difference plots using log2 transformed data. The plots showed a small constant bias and a good agreement between each of two Ki-67 methods and SPF (Figure 4, top center and lower right). However, the correlation between them was moderate: the Kendall coefficient was 0.33 (95% CI 0.26–0.38; *p* < 0.0001) and 0.33 (95% CI 0.26–0.39; *p* < 0.0001) for Method 1 and Method 2, respectively.

### 3.3. Association of Clinic-Pathological Characteristics with Proliferative Indexes SPF and Ki-67

For the analysis of prognostic value of Ki-67 in association with the clinic-pathological characteristic of the patients, only the results of Method 1 were used, as it is routinely used at the Institute of Oncology Ljubljana. 

Tumours with high SPF (≥6.7%) were significantly more likely to be grade 3, ER-negative, stage ≥ pT2, and have lymphatic invasion compared to their counterparts. Similarly, patients with high Ki-67 (≥30%) had a significantly higher proportion of grade 3, ER-negative, ≥stage pT2 tumours, and patients tended to be younger than patients in the low and intermediate Ki-67 groups (Table 1). Interestingly, the SPF and Ki-67 groups were independent of nodal stage. Due to well-known problems with inter-observer variability, different international medical associations recommend different cut-off values for Ki-67. In clinical practice, the biggest dilemma regarding prognosis assessment and choice of systemic therapy is for the Ki-67 intermediate group. Based on the ESMO cut-off, the intermediate group (Ki-67: 11–29%) accounts for 47% of our patients (Table 1). Using the IKWG cut-off, the intermediate group (Ki-67: 6–29%) is even larger (62.8% of all patients, Table S2). Therefore, we focused our analysis on comparing the results of this group with the corresponding low and high proliferation groups.

### 3.4. Treatment and Outcome

The treatment methods and outcomes for the studied patients are presented in Table 3 and Table S3. Two thirds of the patients (66.7%) were treated by mastectomy. The axillary dissection was standard procedure at the time of diagnosis and was performed on all patients included in our retrospective study. One third of the patients (36%) had adjuvant radiation therapy. Half of the patients (50.4%) received adjuvant chemotherapy (CT) (most of them 5-fluorouracil/methotrexate/cyclophosphamide (CMF schedule), and 13.8% anthracyclines), and 56% were treated with endocrine therapy (ET) (tamoxifen and/or goserelin). Patients characterized with high SPF and high Ki-67 were significantly more often treated with CT and less often with ET. The high SPF group had, in addition, a higher proportion of radiation therapy. The adjuvant systemic treatment of ER-positive pre- and postmenopausal patients was significantly different (*p* < 0.001). ET, CT, and CT-ET was delivered in 8.6% vs. 51.2%, 46.4% vs. 12.6%, and 29% vs. 20.3% of cases for pre- vs. post-menopausal patients, respectively. At that time, post-menopausal patients were treated by adjuvant ET routinely, while pre-menopausal patients were not.

The median follow-up time was 26.8 years (range 0.2–30.9). Of total 411 patients, 221 patients (53.1%) had relapsed, and 316 (76.9%) had died (Table 3). The cumulative number of relapse and death events did not differ according to SPF and Ki-67 groups in these long follow-up times; however, the pattern of relapse did (presented in Section 3.5).

### 3.5. Survival Analysis

Kaplan-Meyer curves for RFS and OS are presented in Figure S1. In brief, the median RFS was 8.6 years (95% CI 6.4–10.8) and median OS was 12.3 years (95% CI 9.9–14.6). In studies with a long follow-up period, as in our study, the majority of events after 10 years represent death, not recurrence. Consequently, the evaluation of the TTR may represent the pattern of recurrence more reliably than the RFS.

#### 3.5.1. Time to Recurrences According to SPF and Ki-67 in All Patients

**Figure 6:**
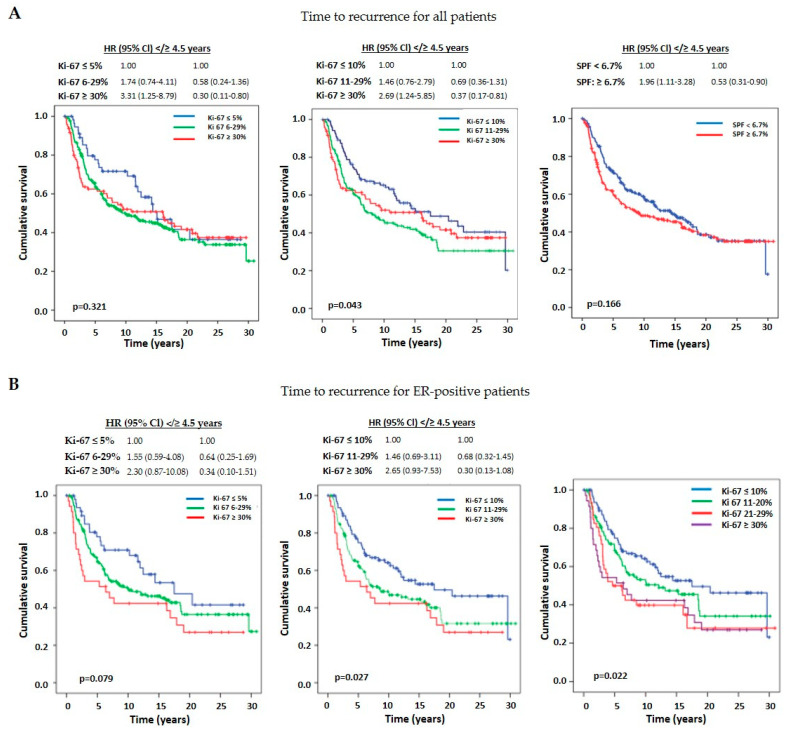
Kaplan–Meier curves showing the time to recurrence for (**A**) all and (**B**) ER-positive patients, stratified by Ki-67 into three groups (using different cut-offs) and by SFP into two groups. According to the curves, high Ki-67 values and high SPF carry a higher risk of early relapse and low Ki-67 values and low SPF carry a risk of long-term (late) recurrence. A cut-off of 5% for Ki-67 better distinguishes the risks of a low and a high group in all patients, and cut-off 10% in ER-positive patients.

Hazard ratios for recurrence for both SPF and Ki-67, when considering all patients, are time-dependent (Figure 6A). In the first 4.5 years after surgery, the hazard ratio of recurrence was 3.31 (*p* = 0.016), 2.69 (*p* = 0.012), and 1.96 (*p* = 0.020) higher in the group with a high proliferation marker than the corresponding group with a low proliferation marker (Ki-67 ≥ 30% vs. ≤5%; Ki-67 ≥ 30% vs. ≤10%, and SPF ≥ 6.7% vs. <6.7%), respectively. After 4.5 years, the hazard ratio of recurrence in each low proliferation group was higher than in the high proliferation group. The intermediate-risk group (6–29%) had a lower hazard ratio in the first 4.5 years compared to the high-risk group, and higher than the lower-risk group. Later on, the hazard ratio was higher in the intermediate-risk group, which is also visible on the estimated survival curves, which cross around 5 years after surgery. The reason for this could be that the mortality in the higher-risk group is greater, thus lowering the hazard ratio of recurrence as a competing event. Interestingly, when using different cut-offs for the intermediate group (Ki-67 = 11–29%), the hazard ratio for recurrence is similar to the high Ki-67 group in the first 4.5 years post-surgery.

#### 3.5.2. Time to Recurrence According to Ki-67 Groups in ER-Positive Patients

**Figure 7:**
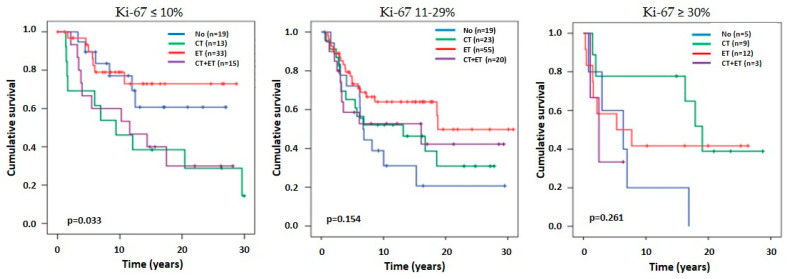
Kaplan-Meier curves of time to recurrence stratified by treatment received in patients with estrogen receptor (ER)-positive disease with 0–3 involved lymph nodes (pN1) for three different Ki-67 risk groups. ET: endocrine therapy; CT: chemotherapy.

We focused most of our interest on analysing the ER-positive cohort (n = 276; 68.7%), with specific emphasis on the Ki-67 intermediate-risk group. TTPs, according to different cut-offs for the intermediate group, are presented on Figure 6B. Our results indicate that that the survival curves of all three Ki-67 groups are separated throughout the follow-up time and do not cross. However, as the rightmost graph shows, for our patients, the survival curve of 21–29% is similar to the Ki-67 ≥ 30% group, suggesting that Ki-67 ≥ 20% could be used as a threshold determining the high Ki-67 group. The results are limited to our sample and should be thoroughly analysed in the future. We also performed an explorative analysis of TTR according to the type of systemic therapy received in the ER-positive pN0-1 subgroup (n = 226) (Figure 7). In the low-risk group (Ki-67 ≤ 10%), patients that were treated with ET alone had lower hazard ratios compared to other treatments (CT or CT-ET; *p* = 0.033). In the intermediate group (Ki-67 = 11–29%), ET and CT-ET showed similar outcomes in TTR (*p* = 0.154). These observations suggest the possibility that the intermediate Ki-67 group might not need treatment with CT, and that CT was even deleterious in the low Ki-67 group; however, further research is needed to confirm these suggestions.

#### 3.5.3. Univariate and Multivariate Survival Analysis

We investigated the prognostic role of SPF and Ki-67, in addition to other clinicopathologic factors, for survival. The summarized analysis for TTR, RFS, and OS is shown in Table 4. For TTR, only nodal stage and lymphovascular invasion were found to be independent prognostic factors in the multivariate Cox regression analysis with proportional hazard assumption. Patients with stage pN2 and pN3 tumours had a 2.2- and 3.75-fold higher hazard ratio for progression, respectively, than patients with stage pN0. Similarly, patients with lymphovascular invasion had a 1.98-fold higher hazard ratio for progression than those without. Hazard ratio for recurrence for Ki-67 was not proportional during the time, as shown on Figure 6A,B. For RFS and OS, age ≥ 50 years, lymphovascular invasion, and nodal stage were independent prognostic factors. Older patients (≥50 years) had a 1.83- and 2.29-fold higher hazard ratio for the event (RFS and OS, respectively) during the 28-year follow-up period compared to patients < 50 years old. Patients with lymphovascular invasion had a 1.51- and 1.41-fold higher hazard ratio for an event (RFS and OS, respectively). Similarly, patients with four or more positive lymph nodes had a 2–3-fold higher hazard ratio for relapse or death compared to patients with negative nodes. Patients with one to three positive lymph nodes did not have a higher hazard ratio for relapse or death than patients with negative lymph nodes.

## 4. Discussion

In our retrospective study of early-stage breast cancer patients, we investigated the agreement and correlation between two different scoring methods for the assessment of proliferative activity determined by IHC detection of the Ki-67 antigen. First, we found excellent agreement between the three pathologists for both scoring methods, visualization across the entire tumour slide (Method 1) and counting on the tumour invasive front (Method 2). Second, there was good agreement between each of IHC methods with SPF, a quantitative flow-cytometric method for estimating cell proliferation. In addition, we found a very good correlation between the two Ki-67 methods, while the correlation between SPF and the two Ki-67 methods was only moderate. For further analysis of the potential prognostic impact of Ki-67, the visual method was used. The risk of recurrence for Ki-67 and SPF was time-dependent. The high proliferation biomarker group (Ki-67 ≥ 30% and SPF ≥ 6.7%) initially had a higher risk of recurrence than the corresponding low proliferation biomarker group, but after 4.5 years, the low proliferation biomarker group had a higher risk of recurrence. In ER-positive patients, the intermediate Ki-67 group (regardless of cut-offs) was clearly separated from the high-risk group in the first 4.5 years and beyond, but was closer to the high Ki-67 group (Ki-67 ≥ 30%) than to the low Ki-67 group (≤5% or 10%). Patients with ER-positive, pN0-1, and intermediate Ki-67 treated with ET had a similar outcome to those treated with CT or CT-ET. In our dataset of ER-positive patients, a cut-off of 20% appeared to be the most appropriate cut-off between the low and high Ki-67 groups.

Ki-67 is a valuable prognostic and predictive marker in breast carcinoma. However, its implementation into routine practice is problematic because of limited reproducibility due to a lack of standardization of procedures and inter-observer variability. Although the IKWG has published recommendations for the assessment of Ki-67 [27,41], different methods are still used [42,43,44,45]. In our present study, the Ki-67 method (Method 1), currently used at the Institute of Oncology Ljubljana, was evaluated and showed almost perfect agreement between pathologists with an ICC value of over 0.90, indicating a very high intra-laboratory reproducibility. This is somewhat surprising, especially in relation to Method 1, as previous studies have shown the visual method to be inaccurate and poorly reproducible [46,47]. This could be due to the fact that the two pathologists specialized in breast pathology are long-time colleagues who work in the same department and often discuss cases together at the discussion microscope. However, the results showed excellent agreement even for the pathology resident with a few months of experience in breast pathology, suggesting that our method is relatively easy to learn. Similarly, Hida et al. reported that visual assessment of Ki67 at a glance with a 5- and 10-grade scale is easy and can help classify luminal-type breast cancers [48,49]. In a recent study, good reproducibility was found between two pathologists visually scoring Ki67 on core biopsies according to IKWG recommendations [50]. However, using the IKWG guidelines, the scoring of one case takes an average of 9–13 min per case, while Method 1 used in our study is much faster. Similar ICC values were reported for visual assessment by del Rosario et al. [45]. Visual “eyeballing” is the most subjective method of analysing Ki-67 proliferative activity; however, it is the most convenient method to use, and some authors claim that it is more accurate than other methods because it is based on an overall impression of a whole slide [51].

We also demonstrated a very strong correlation between the visual method and the quantitative method with the counting of proliferative cells at the invasive front of the tumour (Method 2). Nevertheless, the Bland-Altman plot showed that these two Ki-67 assessment methods cannot be used as substitutes for each other. The range of Ki-67 values was similar for both methods, but the mean and median Ki-67 expression was higher when assessed with Method 2. This result is consistent with other authors, who have reported that Ki-67 methods evaluating the invasive tumour margin yield higher Ki-67 levels than those evaluating the entire slide [52]. In addition, we showed a good agreement between each of two Ki-67 methods and SPF, a quantitative method of assessment of proliferative activity whose prognostic significance was already confirmed in our cohort [15] and also by others [18,19,20,21,22,23,53]. The moderate correlation between methods with higher Ki67 values can be explained by the fact that Ki67 can be detected in all phases of the cell cycle, with the exception of the G0 phase, while SPF only detects cells in the S phase. On the other hand, SPF does not necessarily reflect proliferative activity of malignant components only, but also of other cells present in the cytological sample, such as connective tissue cells and infiltrating lymphocytes [4].

Recently, different automated digital image analysis and artificial intelligence-assisted methods to determine proliferation activity in breast cancer tissue have been proposed. These have led to significant improvement in inter-observer reproducibility [43,45,50,54,55,56,57,58]. Digital image analysis will probably replace manual visual counting in the future, but these techniques are not widely available due to cost reasons and are also time-consuming, as in most cases the pathologist has to label the area to be analysed [43]. Visual methods were found to underestimate Ki67 proliferative activity [44,54]; therefore, cut-off levels should be calibrated when new methods are introduced. The mean and median Ki-67 scores for Method 1 were 22% and 16.3%, respectively, which is in line with other similar studies [45,54].

There is no clear consensus on the cut-off value for Ki-67. On one hand, the Ki-67 score itself is subjective; on the other hand, the tumour can also be heterogeneous, which contributes to the high heterogeneity between observers (in the range of 5–30%). As early as 2001, Spyratos et al. discussed the problem of cut-offs [53]. They concluded that, if Ki-67 is used to exclude patients with slowly proliferating tumours from treatment with chemotherapy, a cut-off of 10% is appropriate. If Ki-67 is used to identify tumours sensitive to chemotherapy, the cut-off should be 25%. In a meta-analysis of 46 studies with 12,155 patients, the cut-off values for high Ki-67 proliferative index were defined according to various criteria, such as mean, median, and tertile distribution, or arbitrarily, and ranged from 3.5% to 34%. This meta-analysis showed that high Ki-67 is associated with worse DFS (HR 1.93) and OS (HR 1.95) [14], but it was not clear whether the proliferation marker Ki-67 provided additional prognostic information compared to the commonly used prognostic factors [59].

In our analysis, we aimed to analyse the outcomes according to the internationally recommended guidelines for Ki-67 cut-offs [27,31]. Both guidelines consider Ki-67 ≥ 30% as a high proliferation value. In our study, 23.3% of patients met the criteria for a high Ki-67 value. However, if the criteria for low KI-67 proliferation according to IKWG (≤5%) and ESMO (≤10%) are chosen, 13.9% and 29.7% of patients met these criteria, respectively. Most cancers (62.8% according to the IKWG, and 47.0% according to the ESMO) were classified in the intermediate Ki-67 group, which is a grey area where we do not know what the prognosis is and how to decide on further treatment. The ESMO criteria were chosen for further analysis; this reduced the size of the ‘undefined’ zone, i.e., the intermediate Ki-67 group.

Analysis of the clinicopathologic parameters showed that patients with high Ki-67 (≥30%) and high SPF (≥6.7%) had a significantly higher proportion of grade 3, ER-negative, and ≥stage pT2 cancers than the corresponding group with low and intermediate Ki-67 and low SPF. High Ki-67 and high SPF were reflected in a worse TTR in the first 4.5 years of follow-up (Figure 6A), and the risk of recurrence was 2–3 times higher than in the groups with low Ki-67 or SPF values. However, later on, the hazard ratio for recurrence in groups with low Ki-67 or low SPF was more than twice those for a group with high Ki-67 or high SPF. For all patients, regardless of ER status, a cut-off of Ki-67 at 5% better differentiates the hazards of a low and a high group. The intermediate Ki-67 group, regardless of the cut-off values, represents a grey area in terms of both prognosis and choice of therapy. This dilemma is particularly important in ER-positive cancers. We evaluated recurrences in this patient group according to Ki-67 groups. The TTR curve of the intermediate Ki-67 group always had an independent course all the time, running between the curves of the high and low Ki-67 groups. In fact, it was more parallel to the high-risk group (Figure 6B). The use of cut-off values for the low-risk group as proposed by ESMO (Ki-67 ≤ 10%) seemed more reasonable, both for prognosis and for deciding the need for chemotherapy for ER-positive patients. However, in our cohort of ER-positive patients, a cut-off of 20% would be even more optimal, as the TTR-Kaplan-Meier curves for the Ki-67 = 20–29% and Ki-67 ≥ 30% groups overlap (Figure 6B). 

Nowadays, Ki-67 assessment should be combined with gene signatures such as Oncotype DX to assess the risk of recurrence and the benefit of chemotherapy. In the West German Study Group PlanB trial, CT was omitted for the Oncotype DX recurrence score (RS) low-risk group (RS 0–11) [60]. Of particular relevance is the ADAPT study with HR+/HER2- cohort, where the role of 2–4 weeks of neoadjuvant ET was investigated in the RS 12–25 group. In patients with ET response, defined as Ki-67 ≤ 10% after short neoadjuvant ET, ET alone was not inferior to CT + ET, and CT could be omitted [61]. In the POETIC trial, postmenopausal patients were receiving a two-week perioperative ET. It was found that patients who already had a low Ki-67 preoperatively had a very favourable outcome during a 5-year follow-up and did not require CT (unless other poor prognostic factors are present, e.g., high nodal stage) [11]. In our ER-positive patients with Ki-67 ≤ 10%, those who received ET alone achieved the most favourable outcomes, while patients who received CT or CT-ET experienced worse outcomes. However, the subset of patients evaluated for treatment outcomes in our study is relatively small. As a result, the interpretation of the Kaplan-Meier survival curves should be approached with caution, considering them as exploratory findings. These results can serve as a basis for generating hypotheses and warrant further investigation in a larger cohort of patients. 

The outcome of our intermediate Ki-67 group was also interesting (Figure 6). There were no significant differences in disease outcome between patients who received ET, CT, or CT-ET. However, the TTR figure shows that the group treated with ET only may have had the best outcome. A very important fact in our study is that only 37.6% of ER+ premenopausal women were prescribed adjuvant ET or CT-ET. However, a further 46.4% were treated with CT, in most cases with the CMF regimen, which carries a significant risk of persistent ovarian suppression, thus reducing the risk of recurrence. This is consistent with the recent report by Gray et al. [62]. In ER-positive premenopausal women having ovarian suppression or ablation, the 15-year risk of recurrence was reduced by 12.1% and 15-year mortality for breast cancer and all-cause mortality were reduced by 8% and 7.2%, respectively. In addition, a sustained long-term benefit of ovarian suppression was demonstrated in the updates of the SOFT and TEXT trials [63].

Findings in our multivariate analysis are consistent with already known prognostic factors for recurrence and death, i.e., nodal stage and lymphovascular invasion. For RFS and OS, age > 50 years was a prognostic factor due to the long follow-up period. Ki-67, however, could not be analysed in the Cox proportional risk assumption model, since we showed its time-dependent hazards in the entire and ER-positive population. To summarize, patients with high Ki-67 and high SPF had a higher early recurrence risk, and patients with low Ki-67 and low SPF had a higher late recurrence risk.

One of the strengths of our study is a large study population with a very long follow-up period. We strictly followed the pre-analytical and analytical requirements for an optimal evaluation of Ki-67. Our pathology department is regularly audited for quality by UQ NEKAS and NORDIC and has proven its worth. Only excision specimens were included in the study and Ki-67 IHC was performed on whole sections. The time from tumour excision to fixation is short in our laboratory, and fixation times do not exceed 72 h. The sections were cut from the paraffin blocks just before staining to avoid prolonged exposure to air. The pathologists had thoroughly discussed and agreed on reporting each Ki-67 assessment method before the study. Since we performed the staining and evaluation of the tissue samples in a single cancer centre, we minimized potential variability. 

In addition to the retrospective design, a limitation could be the exclusion of pathologists from external institutions for the slide evaluation, which we believe would increase the variability and decrease the agreement among pathologists. Similarly, incorporating samples stained at other centres could also lead to these consequences, given the variations in staining procedures among different laboratories. Notably, we followed the St. Gallen recommendations, which advocate interpreting the Ki-67 score based on the local laboratory (median) values [64]. Prolonged storage of the paraffin blocks could be an additional limitation to our study, potentially resulting in antigen degradation and consequently lower Ki-67 scores [65]. However, this is less likely, as the mean and median Ki-67 scores in our study were in the upper range of those published in similar studies [14]. In addition, both IHC-based scoring methods agreed well with the quantitative flow cytometric SFP method. A further limitation is that we do not have information on HER2 status, which was not part of the standard pathology report at the time. We are aware of the fact that HER2 positivity is a significant contributor to early recurrence. Nevertheless, in this study we focused primarily on the evaluation of disease progression in luminal (ER-positive) cancers, with HER2-positive cancers representing only a minority, about 7%, of our cohort. 

Future research will be devoted to new methods for standardising immunohistochemical staining, such as cell line microarray [66], and the use of artificial intelligence in image analysis. Given the remarkable advances in proteomic technology, its use as a quantitative method for assessing tissue expression of biomarkers, including Ki-67, is worth exploring in the future [67]. While all of these new methods are promising, challenges such as cost-effectiveness and limited availability, as well as the need for complex validation studies, remain significant barriers to their widespread use.

## 5. Conclusions

We demonstrated good to excellent inter-observer agreement for two different Ki-67 scoring methods: a semi-quantitative visual assessment of the entire slide, and a quantitative assessment of the tumour invasive front. We confirmed Ki-67 as an important prognostic and predictive biomarker. The investigation of recurrences using different established Ki-67 cut-offs, which classify patients into low and high Ki-67 groups, has shown that a very large group of patients fall into the intermediate group (almost 50% according to the ESMO criteria, and over 60% according to the IKWG criteria). In most cases, such an allocation does not help in assessing prognosis and deciding on therapy options. Our results show that in ER-positive breast cancer with low Ki-67, treatment with ET is the most appropriate. For intermediate Ki-67, ET appears to be as effective as CT-ET in most patients. Recently, gene expression signatures (i.e., the Oncotype Dx test) have been introduced for decision-making between treatment with CT-ET or ET. High Ki-67 is a prognostic factor for early recurrence and serves as a decision factor for chemosensitivity.

In summary, Ki-67 is a simple and inexpensive biomarker. When used in a standardised central pathology assessment, it has clinical utility for assessing prognosis and guiding treatment decision-making in ER-positive cancers.

## Figures and Tables

**Table 1:** Baseline characteristics of patients and tumours in all patients (left) and according S-phase fraction and Method 1 of Ki-67 expression in tumours (right).

Characteristic n (%)	All Patients n = 411 (100)	Low SPF (<6.7%) 219 (47)	High SPF (≥6.7%) 218 (53)	*p*-Value	Low Ki-67 1–10% 122 (29.7)	Ki-67 11–29% 193 (47.0)	High Ki-67 ≥30% 96 (23.3)	*p*-Value
**Age** median (IQR)	58.9 (46.7–67.2)	60.5 (47.7–68.6)	58.3 (46.7–65.4)	0.075	58.9 (47.7–68.3)	60.5 (49.2–67.2)	54.8 (44.4–65.4)	0.042 ^1^ 0.023 ^2^
**Age** n (%)				0.704				**0.087**
<50 years	123 (29.9)	56 (45.5)	67 (54.5)		36 (29.3)	50 (40.6)	37 (30.1)	
≥50 years	288 (70.1)	137 (47.6)	151 (52.4)		86 (29.9)	143 (49.6)	59 (20.5)	
**Histology** n (%)				0.077				**0.010**
NST	350 (85.2)	158 (45.1)	192 (54.9)		94 (26.9)	170 (48.6)	86 (24.6)	
ILC and other	61 (14.8)	35 (57.4)	26 (42.6)		28 (45.9)	23 (37.7)	10 (16.4)	
**Grade** n (%)				**<0.0001**				**<0.0001**
Grade 1	42 (10.2)	33 (78.6)	9 (21.4)		31 (73.8)	11 (26.2)	0 (0)	
Grade 2	170 (41.4)	104 (61.2)	66 (38.8)		76 (44.7)	88 (51.8)	6 (3.5)	
Grade 3	199 (48.4)	56 (28.1)	143 (71.9)		15 (7.6)	94 (47.2)	90 (45.2)	
**LVI** n (%)				**0.011**				0.339
Absent	218 (68.3)	111 (50.9)	107 (49.1)		74 (33.9)	101 (46.3)	43 (19.7)	
Present	191 (31.7)	36 (36.6)	65 (64.4)		26 (25.7)	53 (52.5)	22 (21.8)	
**Tumour stage** n (%)				**0.038**				**0.010**
pT1 (≤20 mm)	154 (37.5)	85 (55.2)	69 (44.8)		55 (35.7)	73 (47.4)	26 (16.9)	
pT2 (>20≤50)	221 (53.8)	93 (22.1)	128 (57.9)		53 (24)	110 (49.8)	58 (26.2)	
pT3 (>50 mm)	35 (8.5)	15 (42.9)	20 (57.1)		13 (37.1)	10 (28.6)	12 (34.3)	
Missing	1 (0.2)							
**Lymph nodes** n (%)				0.260				0.559
pN0 (negative)	227 (55.2)	116 (51.1)	111 (48.9)		63 (27.7)	108 (47.6)	56 (24.7)	
pN1 (1–3 positive)	100 (24.3)	44 (44.0)	56 (56.0)		35 (35.0)	44 (44.0)	21 (21.0)	
pN2 (4–9 positive)	49 (11.9)	20 (40.8)	29 (59.2)		15 (39.6)	26 (53.1)	8 (16.3)	
pN3 (≥10 positive)	35 (8.5)	13 (37.1)	22 (62.9)		9 (25.7)	15 (42.9)	11 (31.4)	
**ER** n (%)				**<0.0001**				**<0.0001**
ER negative	126 (31.3)	38 (30.2)	88 (69.8)		24 (19.0)	44 (35.0)	58 (46.0)	
ER positive	276 (68.7)	15 1 (54.7)	125 (45.3)		96 (34.8)	145 (52.5)	35 (12.7)	

ER: Estrogen receptor; IQR: interquartile range, LVI: lymphovascular invasion, NST: invasive carcinoma of no special type, S-phase fraction: SPF. ^1^ low vs. high Ki-67, ^2^: intermediate vs. high-Ki-67. Bolded values in the table represent statistically significant results.

**Table 2:** Intra-class correlation estimates and their 95% confidence intervals based on mean rating (k = 3), absolute agreement, and two-way random effects. Single-value measures were used for interpretation.

	Intraclass Correlation	95% Confidence Interval		F-Test with True Value 0			
	Single Measures	Lower Bound	Upper Bound	Value	df1	df2	Sig.
Method 1	0.962	0.926	0.978	110.934	410	410	0.000
Method 2	0.946	0.888	0.969	79.217	410	820	0.000

**Table 3:** Main treatment modalities and outcome in patients in relation to Ki-67 and SPF values.

Type of Treatment	All Patients (n = 411)	Low SPF (<6.7%) (n = 193)	High SPF (≥6.7%) (n = 218)	*p*-Value	Low Ki-67 1–10% 122 (29.7)	Intermediate Ki-67 11–29% 193 (47.0)	High Ki-67 ≥30% 96 (23.3)	*p*-Value
**Type of surgery**				0.780				0.864
Mastectomy	274 (66.7)	130 (47.4)	144 (52.6)		83 (30.3)	129 (47.1)	62 (22.6)	
BCS	137 (33.3)	63 (46)	74 (54)		39 (28.5)	64 (46.7)	34 (24.8)	
**Adjuvant RT**	148 (36)	57 (38.5)	91 (61.5)	**0.010**	42 (28.4)	65 (43.9)	41 (27.7)	0.293
**Adjuvant ET**	224 (55.7)	116 (51.8)	108 (42.8)	**0.001**	69 (30.8)	115 (51.3)	40 (17.9)	**<0.0001**
ER negative	50 (22.3)	16 (32)	34 (68)		9 (18)	20 (40)	21 (42)	
ER positive	174 (77.7)	100 (57.5)	74 (42.5)		60 (34.5)	95 (54.6)	19 (10.9)	
**Adjuvant CMF**	207 (50.4)	86 (41.5)	121 (58.5)	**0.001**	54 (26.5)	92 (45.1)	58 (28.4)	**<0.0001**
ER negative	84 (41.2)	23 (27.4)	62 (72.6)		15 (17.9)	26 (31)	43 (51.1)	
ER positive	120 (58.8)	62 (51.7)	58 (48.3)		39 (32.5)	66 (55)	15 (12.5)	
**Events**								
Relapse	221 (53.1)	99 (44.8)	122 (55.2)	0.213	56 (25.3)	112 (50.2)	53 (24.0)	0.104
Death	316 (76.9)	151 (47.8)	165 (52.2)	0.540	92 (29.1)	155 (49.1)	69 (21.8)	0.249

CMF: chemotherapy consisting of cyclophosphamide 5-fluorouracil and methotrexate; BCS: breast conserving surgery; ET: endocrine therapy; RT: radiation therapy. Bolded values in the table represent statistically significant results.

**Table 4:** Results of univariable and multivariable analysis of prognostic factors for time to recurrence, relapse-free, and overall survival. NST: invasive carcinoma of no special type, ILC: invasive lobular carcinoma, ER: estrogen receptors, LVI: lymphovascular invasion, SFP: S-phase fraction.

	Time to Progression				Relapse-Free Survival				Overall Survival			
	Univariable HR (95%CI)	*p*-Value	Multivariable HR (95% CI)	*p*-Value	Univariable HR (95% CI)	*p*-Value	Multivariable HR (95% CI)	*p*-Value	Univariable HR (95% CI)	*p*-Value	Multivariable HR (95% CI)	*p*-Value
Age < 50 years	1.00	0.214	**/**	/	1.00	<0.0001	1.00	<0.0001	1.00	<0.0001	1.00	<0.0001
Age ≥ 50 years	1.20 (0.90–1.61)				1.80 (1.40–2.32)		1.83 (1.36–2.47)		2.25 (1.72–2.94)		2.29 (1.66–3.15)	
Histology												
NST	1.00	0.506	/	/	1.00	0.753	/	/	1.00	0.592	/	/
ILC + other	1.13 (0.79–1.60)				1.05 (0.78–1.41)				0.92 (0.67–1.25)			
Grade												
Grade 1	1.00	0.082	1.00	0.518	1.00	0.206	/	/	1.00	0.077	1.00	0.397
Grade 2	1.50 (0.89–2.52)	0.126	1.23 (0.63–2.41)	0.539	1.41 (0.96–2.06)	0.077			1.55 (1.04–2.30)	0.032	1.27 (0.78 (2.09)	0.340
Grade 3	1.75 (1.05–2.91)	0.031	1.41 (0.72–2.74)	0.316	1.29 (0.89–1.88)	0.180			1.55 (1.04–2.29)	0.030	1.40 (0.85–2.30)	0.185
Tumor stage												
pT1 (≤20 mm)	1.00	<0.0001	1.00	0.898	1.00	<0.0001	1.00	0.489	1.00	0.001	1.00	0.772
pT2 (21–50 mm)	1.49 (1.11–1.99)	0.008	1.04 (0.74–1.48)	0.809	1.42 (1.13–1.80)	0.003	1.07 (0.81–1.41)	0.635	1.37 (1.08–1.74)	0.010	1.00 (0.75–1.33)	0.994
pT3 (>50 mm)	2.73 (1.74–4.28)	<0.0001	0.92 (0.50–1.70)	0.797	2.60 (1.75–3.82)	<0.0001	1.39 (0.81–2.38)	0.232	1.98 (1.33–2.95)	0.001	1.20 (0.71–2.02)	0.505
Nodal stage												
pN0 (negative)	1.00	<0.001	1.00	<0.0001	1.00	<0.0001	1.00	<0.0001	1.00	<0.0001	1.00	<0.0001
pN1 (1–3 positive)	1.19 (0.86–1.65)	0.294	1.23 (0.83–1.82)	0.310	1.05 (0.80–1.36)	0.747	1.81 (0.86–1.62)	0.301	1.04 (0.79–1.37)	0.792	1.31 (0.95–1.81)	0.102
pN2 (4–9 positive)	2.29 (1.56–3.37)	<0.0001	2.20 (1.40–3.46)	0.001	2.33 (1.68–3.24)	<0.0001	2.13 (1.47–3.09)	<0.0001	2.69 (1.92–3.77)	<0.0001	2.72 (1.85–4.00)	<0.0001
pN3 (≥10 positive)	3.98 (2.61–6.08)	<0.0001	3.75 (2.20–6.37)	<0.0001	43.21 (2.20–4.68)	<0.0001	2.52 (1.57 (4.07)	<0.0001	3.35 (2.92–4.89)	<0.0001	2.84 1.79–4.53)	<0.0001
LVI												
No	1.00	<0.0001	1.00	<0.0001	1.00	<0.0001	1.00	0.003	1.00	0.007	1.00	0.022
Yes	2.37 (1.74–3.23)		1.98 (1.42–2.76)		1.70 (1.31–2.21)		1.54 (1.16–2.04)		1.45 (1.1–1.89)		1.40 (1.05–1.88)	
ER negative	1.00	0.917	/	/	1.00	0.571	/	/	1.00	0.729	/	/
ER positive	0.99 (0.74–1.31)				1.07 (0.84–1.36)				1.04 (0.82–1.33)			
Low SPF (<6.7%)	1.00	0.232	/	/	1.00	0.530	/	/	1.00	0.409	/	/
High SPF (≥6.7%)	1.18 (0.90–1.53)				1.07 (0.86–1.33)				1.10 (0.88–1.37)			
Ki-67												
Low (≤5%)	1.00	0.324	/	/	1.00	0.623	/	/	1.00	0.760	/	/
Intermediate (6–29%)	1.38 (0.90–2.12)	0.136			1.08 (0.78–1.49)	0.649			1.05 (0.76–1.46)	0.760		
High (≥30%)	1.35 (0.84–2.18)	0.214			0.95 (0.65–1.38)	0.784			0.95 (0.65–1.39)	0.794		

## Data Availability

The data presented in this study are available on reasonable request from the corresponding author. The data are not publicly available due to data protection regulations of the participating medical institutions.

## Supplementary Materials

The following supporting information can be downloaded at: https://www.mdpi.com/article/10.3390/cancers16071405/s1, Figure S1: Survival curves for relapse-free survival and overall survival with corresponding median survival; Table S1: Summary statistics of log2-transformed measurements for each of two methods (M1 and M2) of Ki-67 assessment by three different pathologists; Table S2: Baseline characteristics of patient and tumour in all patients and according S-phase fraction (SPF) and Method 1 of Ki-67 expression; Table S3: Main treatment modalities in patients.
