# Supplementary material for: Clinical and Analytical Validation of Two Methods for Ki-67 Scoring in Formalin Fixed and Paraffin Embedded Tissue Sections of Early Breast Cancer
Source: Cancers (Basel). 2024 Apr 3;16(7):1405. doi: 10.3390/cancers16071405 (PMC11011015; doi:10.3390/cancers16071405)
Supplement: Supplementary file 1 [file cancers-16-01405-s001.zip › cancers-2926663-supplementary.pdf]

Table S1. Summary statistics of log2-transformed measurements for each of three methods (M1, M2 and M3) of Ki-67 assessment by three different pathologists

Method	Pathologist	Min	Q1	Median	Mean	Q3	Max	SD	N. of missing observations
M1	1	0.14	3.34	4.18	4.03	4.91	6.46	1.26	0
M1	2	0.14	3.34	4.18	4.06	4.92	6.57	1.29	0
M1	3	0.14	3.02	3.92	3.76	4.65	6.41	1.34	0
M2	1	0.14	3.60	4.32	4.30	5.05	6.57	1.13	0
M2	2	0.14	3.60	4.33	4.36	5.17	6.60	1.14	0
M2	3	0.14	3.19	4.01	4.00	4.92	6.48	1.23	0
M3	1	1.63	3.92	4.65	4.49	5.25	6.41	1.11	336
M3	2	1.63	3.84	4.70	4.57	5.50	6.43	1.15	339
M3	3	1.07	3.87	4.47	4.37	5.13	6.34	1.09	338

Table S2. Baseline characteristics of patient and tumor in all patients (left part of table) and according S-phase fraction (SPF) and method 1 of Ki-67 expression in tumor (right part of table).
NST = invasive carcinoma of no special type; LVI = lymphovascular invasion; IQR = interquartile range

**Characteristic** n (%)	**All patients** n=411 (100)	**Low SPF (<6.7%)** n=193 (47)	**High SPF (≥6.7%)** n=218 (53)	**p-value**	**Low Ki-67 1-5%** n=57 (13.9)	**Ki-67 6-29%** n=258 (62.8)	**High Ki-67 ≥30%** n=96 (23.3)	**p-value**
**Age** median (IQR)	58.9 (46.7-67.2)	60.5 (47.7-68.6)	58.3 (46.7-65.4)		60.1 (50.2-68.1)	60.5 (50.3-60.0)	54.8 (44.4-65.4)	
**Age** n **(%)**				0.704				0.074
<50 years	123 (29.9)	56 (45.5)	67 (54.5)		13 (10.6)	71 (59.2)	37 (30.8)	
≥50 years	288 (70.1)	137 (47.6)	151 (52.4)		44 15.3)	185 (64.2)	59 (21.1)	
**Histology** n (%)				0.077				<0.0001
NST	350 (85.2)	158 (45.1)	192 (54.9)		38 (10.9)	226 (64.6)	86 (24.6)	
ILC and other	61 (14.8)	35 (57.4)	26 (42.6)		19 (31.1)	32 (52.5)	10 (16.4)	
**Grade** n (%)				<0.0001				<0.0001
Grade 1	42 (10.2)	33 (78.6)	9 (21.4)		21 (50.0)	21 (50.0)	0 (0)	
Grade 2	170 (41.4)	104 (61.2)	66 (38.8)		32 (18.8)	132 (77.6)	6 (3.6)	
Grade 3	199 (48.4)	56 (28.1)	143 (71.9)		4 (2.0)	105 (52.8)	90 (45.2)	
**LVI** n (%)				0.011				0.495
Absent	218 (68.3)	111 (50.9)	107 (49.1)		37 (17.0)	138 (63.3)	43 (19.7)	
Present	191 (31.7)	36 (36.6)	65 (64.4)		12 (11.9)	67 (66.3)	22 (21.8)	
**Tumor stage** n (%)				0.038				0.014
pT1 (≤20mm)	154 (37.5)	85 (55.2)	69 (44.8)		28 (18.2)	100 (64.9)	26 (16.9)	
pT2 (>20≤50)	221 (53.8)	93 (22.1)	128 (57.9)		22 (10.0)	141 (63.8)	58 (26.2)	
pT3 (>50 mm)	35 (8.5)	15 (42.9)	20 (57.1)		7 (20.0)	16 (45.7)	12 (34.3)	
Missing	1 (0.2)							
**Lymph nodes** n (%)				0.260				0.355
pN0 (negative)	227 (55.2)	116 (51.1)	111 (48.9)		25 (11.0)	146 (64.3)	56 (24.7)	
pN1 (1-3 positive)	100 (24.3)	44 (44.0)	56 (56.0)		19 (19.0)	60 (60.0)	21 (21.0)	
pN2 (4-9 positive)	49 (11.9)	20 (40.8)	29 (59.2)		8 (16.3)	33 (67.3)	8 (16.3)	
pN3 (≥10 positive)	35 (8.5)	13 (37.1)	22 (62.9)		5 (14.3)	19 (54.3)	11 (31.4)	
**Estrogen receptor (ER)** n (%) (9)				<0.0001				<0.0001
ER negative	126 (31.3)	38 (30.2)	88 (69.8)		7 (5.6)	61 (48.4)	58 (46.0)	
ER positive	276 (68.7)	15 1 (54.7)	125 (45.3)		49 (17.8)	192 (69.6)	35 (12.7)	

Table S3. Main treatment modalities in patients.
CMF: chemotherapy consisting of cyclophosphamide, 5-fluorouracil and methotrexate; RT: radiation therapy; ET: endocrine therapy; Cht: chemotherapy

**Type of treatment**	**All patients** (n=441)	**Low SPF (<6.7%)** (n=193)	**High SPF (≥6.7%)** (n=218)	**p-value**	**Low Ki-67 1-5%** (n=57)	**Intermediate Ki-67 6-29%** (n=258)	**High Ki-67 ≥30%** (n=96)	**p-value**
**Type of surgery**				0.780				0.513
Mastectomy	274 (66.7)	130 (47.4)	144 (52.6)		35 (12.8)	177 (64.6)	62 (22.6)	
BCS	137 (33.3)	63 (46)	74 (54)		22 (16.1)	81 (59.1)	34 (24.8)	
**Adjuvant RT**				0.010				0.078
No	263 (64.0)	136 (51.7)	127 (48.3)		43 (16.3)	165 (62.8)	55 (20.9)	
Yes	148 (36.0)	57 (38.5)	91 (61.5)		14 (9.5)	93 (62.8)	41 (27.7)	
**Adjuvant ET**				0.029				0.011
No	181 (44.0)	74 (40.9)	107 (59.1)		19 (10.5)	108 (59.7)	54 (29.8)	
Yes	230 (56.0)	119 (51.7)	111 (48.3)		38 (16.5)	150 (65.2)	42 (18.3)	
**Adjuvant Cht**				0.027				0.040
No	204 (49.6)	107 (52.5)	97 (47.5)		32 (15.7)	135 (66.2)	37 (18.1)	
Yes	207 (50.4)	86 (41.5)	121 (58.5)		25 (12.1)	123 (59.4	59 (28.5)	
**Events**								
Relapse	221 (53.1)	99 (44.8)	122 (55.2)	0.213	25 (11.3)	143 (64.7)	53 (24.0)	0.270
Death	316 (76.9)	151 (47.8)	165 (52.2)	0.540	44 (13.9)	203 (64.2)	69 (21.8)	0.401

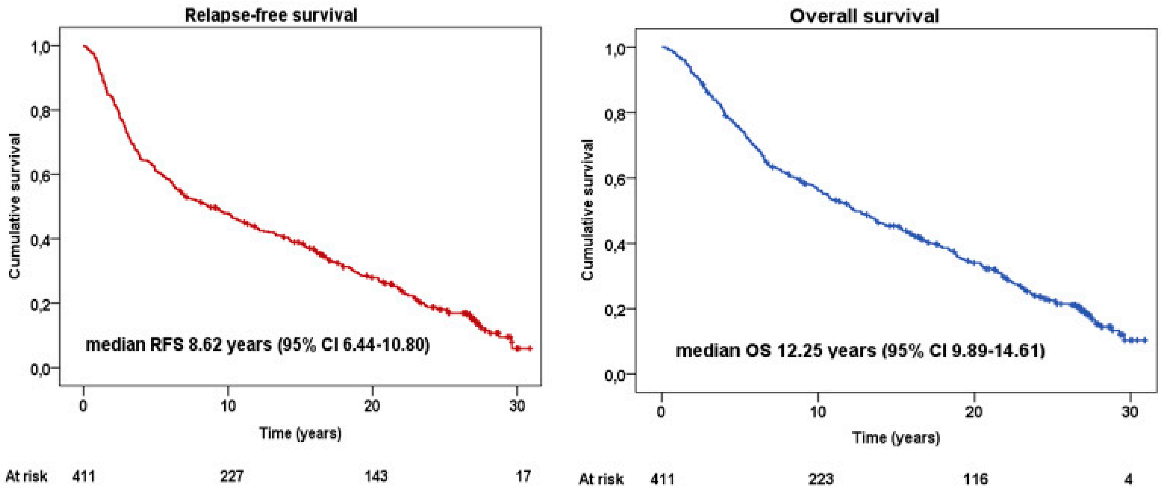

Figure S1. Survival curves for relapse-free survival and overall survival with corresponding median survival.
